# Post-breastfeeding stress response and breastfeeding self-efficacy as modifiable predictors of exclusive breastfeeding at 3 months postpartum: a prospective cohort study

**DOI:** 10.1186/s12884-020-03431-8

**Published:** 2020-11-25

**Authors:** Mie Shiraishi, Masayo Matsuzaki, Shoko Kurihara, Maki Iwamoto, Mieko Shimada

**Affiliations:** 1 Department of Children and Women’s Health, Division of Health Sciences, Graduate School of Medicine, Osaka University, 1-7, Yamadaoka, Suita, Osaka, 565-0871 Japan; 2 Graduate Program of Midwifery, Dokkyo Medical University, Tochigi, Japan

**Keywords:** Breastfeeding, Postpartum period, Self-efficacy, Stress response

## Abstract

**Background:**

The rate of exclusive breastfeeding at 3 months postpartum is only 50% in Japan. In order to increase this rate, we aimed to examine modifiable factors related to exclusive breastfeeding at 3 months postpartum by focusing on breastfeeding-related and psychosocial variables at 1 month postpartum.

**Methods:**

This prospective cohort study was conducted at a secondary medical care center in Osaka, Japan from February 2017 to October 2018. Demographic variables, infant feeding modality, breastfeeding-related variables, and psychosocial variables were obtained using questionnaires at 1 month postpartum. Daytime salivary cortisol levels before and after breastfeeding at 1 month postpartum were measured as a biological marker for stress responses associated with breastfeeding. Each infant’s feeding modality was re-assessed at 3 months postpartum. Multiple logistic regression analyses were performed to examine factors affecting exclusive breastfeeding at 3 months postpartum.

**Results:**

Of the 104 participants, 61 reported exclusive breastfeeding at 3 months postpartum. The following factors were significantly associated with exclusive breastfeeding at 3 months postpartum: multiparity (adjusted odds ratio, 95% confidence interval: 11.13, 2.08–59.59), having a university degree (5.25, 1.04–26.53), no plan to return to work by 6 months postpartum (0.02, 0.00–0.46), and exclusive breastfeeding (42.84, 6.05–303.52), lower cortisol level after breastfeeding (0.00, 0.00–0.02), and higher breastfeeding self-efficacy scale score (1.07, 1.00–1.14) at 1 month postpartum. In parity-specific analyses, exclusive breastfeeding (25.33, 4.75–134.98) and lower cortisol level after breastfeeding (0.00, 0.00–0.21) at 1 month postpartum in primiparous women, and lower cortisol level after breastfeeding (0.00, 0.00–0.94), higher breastfeeding self-efficacy score (1.18, 1.05–1.32), and absence of breast complications (0.09, 0.01–0.82) at 1 month postpartum in multiparous women were associated with exclusive breastfeeding at 3 months postpartum.

**Conclusions:**

Stress levels after breastfeeding, breastfeeding self-efficacy, and the presence of breast complications could be modifiable factors associated with subsequent exclusive breastfeeding. Further research is needed to examine whether approaches to reducing breastfeeding-related stress, improving breastfeeding self-efficacy, and preventing breast complications during lactation are effective to increase exclusive breastfeeding practices.

## Background

The World Health Organization recommends exclusive breastfeeding for at least 6 months after childbirth for optimal growth, development, and well-being of children [1]. However, in Japan, the rate of exclusive breastfeeding in the first 3 months postpartum ranged from only 51 to 55% in 2015 [2]. According to a national survey in Japan [3], 41.7% of women planned exclusive breastfeeding during pregnancy, and 49.3% stated they would breastfeed if possible; that is, more than 90% expressed a desire to breastfeed. At 1 month postpartum, 67.6% of the women who planned exclusive breastfeeding, and 40.7% of those who stated that they would breastfeed if possible were exclusively breastfeeding. Although many pregnant women have a desire to breastfeed, actual postpartum feeding modality does not necessarily conform to antenatal intent.

Exclusive breastfeeding practice is related to demographic, physiological, and psychological factors. Demographic factors including age, parity, and education level have consistently characterized exclusive breastfeeding mothers [4–6]. Mothers who did not experience breast complications such as nipple pain, reported practicing exclusive breastfeeding more than those with breast complications [7]. Such physiological barriers might increase stress levels and cause women to discontinue breastfeeding sooner [8, 9]. In addition, psychosocial factors, including breastfeeding-related self-efficacy, stress levels, and family support, might also predict exclusive breastfeeding practice. According to Otsuka et al. [10], low breastfeeding self-efficacy is associated with the perception of insufficient milk, which may lead to milk supplementation [3]. In a cross-sectional study, higher subjective child-related stress responses were reported in women who were partially breastfeeding than in those who were exclusively breastfeeding [11]. Meanwhile, an association between breastfeeding-related stress and subsequent breastfeeding practice has been not clarified, although psychological stress is known to decrease suckling-induced oxytocin release [12]. Kaneko et al. [13] reported that husband support, such as advice on child-rearing, is associated with exclusive breastfeeding. The effects of these physiological and psychosocial factors on subsequent exclusive breastfeeding practice have not been longitudinally examined.

To meet the breastfeeding expectations of women and promote exclusive breastfeeding practices, we aimed to examine modifiable factors at 1 month postpartum related to exclusive breastfeeding at 3 months postpartum. In particular, we focused on breastfeeding-related and psychosocial variables such as breast complications, breastfeeding self-efficacy, stress levels, depressive symptoms, bonding, and family support. Breastfeeding-related stress levels were assessed using salivary cortisol measurement, as objective stress markers can better reflect immediate stress responses than subjective assessment. A clearer understanding of these associations may help healthcare professionals develop important interventions to promote exclusive breastfeeding.

## Methods

### Participants and setting

This prospective cohort study was a secondary analysis of a longitudinal study. The longitudinal study from the third trimester to 5 months postpartum was conducted at a secondary medical care center in Osaka, Japan from February 2017 to October 2018. The research hospital was not a baby-friendly hospital as defined by the World Health Organization/United Nations International Children’s Emergency Fund. Healthy Japanese women at 34–36 weeks’ gestation who visited the hospital for medical checkup were recruited. The following women were excluded: those aged < 20 years, those with inadequate Japanese literacy, and those with psychiatric diseases. All participants provided written informed consent prior to the baseline investigation. Of the investigation data available at the third trimester, a few days after childbirth, and 1, 3, and 5 months postpartum in the longitudinal study, we used data from the third trimester and at 1 and 3 months postpartum. The investigations at 1 and 3 months postpartum were conducted by mail. We did not use data at a few days postpartum as the breastfeeding-related stress levels in this period seemed unstable. This was because many women have breast complications, such as nipple pain and breast engorgement, and they are not accustomed to the breastfeeding process. In addition, we did not use data at 5 months postpartum because of the high dropout rate. We also used data on delivery outcomes from the participants’ medical charts.

### Variables

The original questionnaire distributed to the mothers at 34–36 weeks’ gestation contained questions on age, parity, education level, height, and pre-pregnancy weight. After childbirth, we obtained information about the delivery mode, blood loss during delivery, and infant’s birth weight from the medical charts. At 1 month postpartum, we mailed a questionnaire and a box containing two tubes for saliva sampling and icepack to the participants. The participants collected 1.0-mL saliva samples twice (before and after breastfeeding of 11:00–15:00). Salivary cortisol levels were measured as an indicator of the physiological and psychological responses associated with breastfeeding. The sampling time 11:00–15:00 was chosen because cortisol levels are comparatively stable in the circadian rhythm during this time [14]. The participants were asked not to eat or drink in the 15 min before saliva sampling. After the sampling, they packed the two saliva samples and the icepack into the box and mailed them to the research university using a frozen delivery service. Thereafter, the saliva samples were stored at − 30 °C until analysis. The sampling method was based on the method used by Kusaka et al. [15]. The salivary cortisol levels were measured using a Cortisol Salivary Immunoassay Kit (Salimetrics, LLC, CA, USA).

The questionnaire at 1 month postpartum contained questions on infant feeding modality, breast complications (nipple pain, cracked nipples, nipple shape, breast induration, and breast inflammation), the Japanese version of the Breastfeeding Self-Efficacy Scale Short Form (BSES-SF) [10], the Japanese version of the Perceived Stress Scale-10 (PSS-10) [16], the Japanese version of the Edinburgh Postnatal Depression Scale (EPDS) [17], the Japanese version of the Postpartum Bonding Questionnaire (J-PBQ) [18], and support from husbands or parents. Infant feeding modality was selected from among exclusive breastfeeding, partial breastfeeding, or formula feeding.

The BSES-SF was developed to assess a person’s belief and confidence in their ability to breastfeed [10, 19]. The breastfeeding self-efficacy theoretical framework developed by Dennis [20] was based on Bandura’s social cognitive theory [21]. This scale consists of 14 items that require 5-point Likert scale responses ranging from “not at all confident” to “very confident” (total, 14–70 points). Higher scores indicate a higher level of self-efficacy. Cronbach’s α was set at 0.849.

The PSS-10 was used to assess the degree to which situations in one’s life are appraised as stressful [16, 22]. This scale consists of 10 items requiring 5-point Likert scale responses (total, 0–40 points). Higher scores indicate higher stress levels. Cronbach’s α was set at 0.854.

The EPDS assesses whether a person had depressive symptoms within the preceding 7 days [17, 23]. This scale is a 10-item self-reported screening tool for perinatal depression with a total score between 0 and 30. In Japan, a postpartum woman with a score > 8 points is considered to have depressive symptoms [17]. Cronbach’s α was set at 0.792.

The J-PBQ consists of 14 items, and is used to assess whether participants have bonding disorders [18]. The J-PBQ was developed based on the original PBQ by Brockington et al. [24], which consists of 25 items. The J-PBQ has 6-point Likert scale responses ranging from “never” to “always” (total, 0–70 points). Higher scores indicate a greater bonding disorder. Cronbach’s α was set at 0.813.

At 3 months postpartum, we re-assessed each infant’s feeding modality.

### Statistical analysis

Student’s *t*-test, Mann-Whitney *U* test, chi-square test, or Fisher’s exact test was used to compare participant characteristics between women who were exclusively breastfeeding and those who were not, at 3 months postpartum. Multiple logistic regression analyses were performed to examine factors associated with exclusive breastfeeding at 3 months postpartum. Variables with *p* values < 0.10 for binary analyses and demographic variables (age, parity, and education level) identified as factors associated with breastfeeding in previous studies [4, 12] were entered as independent variables into the multiple logistic regression models. Independent variables in the regression final model were determined using backward elimination (Wald). These analyses were conducted according to parity. All differences with a two-sided *p* value < 0.05 were considered significant. Statistical analyses were performed using Statistical Package for Social Sciences v. 24.0® (IBM Corp, Armonk, NY, USA).

## Results

Of the 349 healthy women at 34–36 weeks’ gestation who were asked to participate in the research, 269 provided written informed consent. Among them, 70 were excluded due to consent withdrawal by 1 month postpartum (*n* = 66), psychological condition due to after-effects of earthquake (*n* = 2), severe infant condition (*n* = 1), and isolation by infection (*n* = 1). Thus, we asked 199 (74.0%) women to answer questionnaires and collect saliva samples at 1 month postpartum. Of these 199 women, 41 did not submit the questionnaires and salivary samples. Of the 158 (58.7%) kits received, 43 were excluded due to incomplete data (*n* = 33), answering questionnaires over 60 days after childbirth (*n* = 5), inadequate salivary sampling (*n* = 4), and formula feeding (*n* = 1). Thus, at 3 months postpartum, 115 (42.8%) women were asked to answer a questionnaire about infant feeding modality. Of these, 11 women did not return the questionnaire. Finally, a total of 104 (38.7%) women provided complete baseline data, answered further questionnaires, and provided daytime saliva samples. No differences in demographic variables and infant feeding modality and salivary cortisol levels at 1 month postpartum were found between the dropouts and participants who completed all investigations.

Table 1 shows the summary of participant characteristics. Of the 104 women, 61 (58.7%) reported exclusive breastfeeding at 3 months postpartum; 33 (31.7%), partial breastfeeding; and 10 (9.6%), formula feeding. The analyses were conducted according to parity, as parity was strongly associated with breastfeeding practice and other variables.

**Table 1:** Participant characteristics

	All participants (*n* = 104)	Exclusive breastfeeding at 3 months postpartum (*n* = 61)	Non-exclusive breastfeeding at 3 months postpartum (*n* = 43)	*P*	Primiparas (*n* = 58)			Multiparas (*n* = 46)		
					Exclusive breastfeeding at 3 months postpartum (*n* = 26)	Non-exclusive breastfeeding at 3 months postpartum (*n* = 32)	*p*	Exclusive breastfeeding at 3 months postpartum (*n* = 35)	Non-exclusive breastfeeding at 3 months postpartum (*n* = 11)	*p*
	Mean ± SD or n (%)	Mean ± SD or n (%)	Mean ± SD or n (%)		Mean ± SD or n (%)	Mean ± SD or n (%)		Mean ± SD or n (%)	Mean ± SD or n (%)	
Age [year]	34.0 ± 4.7	33.4 ± 4.6	34.9 ± 4.2	0.103	32.0 ± 5.1	34.8 ± 4.2	0.025	34.5 ± 4.0	35.3 ± 4.6	0.597
Parity: Multipara [n (%)]	58 (55.8)	35 (57.4)	11 (25.6)	0.001^a^						
Education level [n (%)]				0.354^a^			0.311^a^			0.103^a^
University or above	54 (51.9)	34 (55.7)	20 (46.5)		18 (69.2)	18 (56.3)		16 (45.7)	2 (18.2)	
Others	50 (48.1)	27 (44.2)	23 (53.5)		8 (30.8)	14 (43.8)		19 (54.3)	9 (81.8)	
Working	72 (69.2)	42 (68.9)	30 (69.8)	0.921^a^	7 (26.9)	9 (28.1)	0.919^a^	12 (34.3)	4 (36.4)	1.000^b^
Planning to return to work by 3 months postpartum	6 (5.8)	1 (1.6)	5 (11.6)	0.079^b^	0(0.0)	3 (9.4)	0.245^b^	1 (2.9)	2 (18.2)	0.138^b^
Planning to return to work by 6 months postpartum	9 (8.7)	2 (3.3)	7 (16.3)	0.031^b^	0(0.0)	5 (15.6)	0.058^b^	2 (5.7)	2 (18.2)	0.238^b^
Unplanned pregnancy	21 (20.2)	13 (21.3)	8 (18.6)	0.735^a^	7 (26.9)	5 (15.6)	0.291^a^	6 (17.1)	3 (27.3)	0.664^b^
Height [cm]	158.5 ± 5.6	157.8 ± 5.8	159.5 ± 5.3	0.145	159.5 ± 5.4	159.6 ± 5.3	0.903	156.6 ± 5.9	159.1 ± 5.4	0.234
Pre-pregnancy weight [kg]	52.8 ± 7.4	52.4 ± 6.8	53.3 ± 8.2	0.552	52.2 ± 5.2	52.0 ± 6.8	0.922	52.6 ± 7.9	56.9 ± 10.9	0.152
Pre-pregnancy body mass index [kg/m^2^]	21.0 ± 2.6	21.0 ± 2.5	20.9 ± 2.8	0.830	20.5 ± 2.0	20.4 ± 2.2	0.781	21.0 ± 2.5	20.9 ± 2.8	0.830
Delivery mode										
Natural childbirth [n (%)]	60 (57.7)	41 (67.2)	19 (44.2)	0.019^a^	14 (53.8)	12 (37.5)	0.213^a^	27 (77.1)	7 (63.6)	0.374^a^
Induction and augmentation of labour [n (%)]	14 (13.6)	6 (10.0)	8 (18.6)	0.209^a^	6 (23.1)	7 (21.9)	0.913^a^	0(0.0)	1 (9.1)	0.244^b^
Instrumental vaginal birth [n (%)]	9 (8.7)	3 (4.9)	6 (14.0)	0.157^a^	1 (3.8)	5 (15.6)	0.209^b^	2 (5.7)	1 (9.1)	1.000^b^
Planned Cesarean Section [n (%)]	12 (11.5)	6 (9.8)	6 (14.0)	0.547^b^	2 (7.7)	4 (12.5)	0.681^b^	4 (11.4)	2 (18.2)	0.619^b^
Emergency Cesarean Section [n (%)]	8 (7.7)	4 (6.6)	4 (9.3)	0.715^b^	3 (11.5)	4 (12.5)	1.000^b^	1 (2.9)	0(0.0)	1.000^b^
Gestational age at delivery [weeks]	39.2 ± 1.1	39.1 ± 1.1	39.3 ± 1.2	0.311	39.3 ± 1.2	39.5 ± 1.3	0.715	39.1 ± 1.1	39.3 ± 1.2	0.866
Blood loss during delivery [g]	542 ± 357	474 ± 268	639 ± 440	0.032	564 ± 285	667 ± 464	0.325	474 ± 268	639 ± 440	0.233
Infant’s birth weight [g]	3083 ± 332	3061 ± 314	3115 ± 357	0.424	3030 ± 358	3084 ± 368	0.576	3061 ± 314	3115 ± 357	0.234

*SD* Standard deviation

Student’s *t*-test. ^a^Chi-square test. ^b^Fisher’s exact test

The associations between breastfeeding-related and psychosocial variables at 1 month postpartum and exclusive breastfeeding at 3 months postpartum are shown in Table 2. Of the women who were not exclusively breastfeeding at 1 month postpartum, 14 (26.9%) changed to exclusive breastfeeding at 3 months postpartum. In contrast, 5 (10.4%) women who were exclusively breastfeeding at 1 month postpartum changed to partial breastfeeding by 3 months postpartum. Forty-five (43.3%) women had higher salivary cortisol levels after breastfeeding than before.

**Table 2:** Breastfeeding-related variables and psychosocial variables at 1 month postpartum

	All participants (*n* = 104)	Exclusive breastfeeding at 3 months postpartum (*n* = 61)	Non-exclusive breastfeeding at 3 months postpartum (*n* = 43)	*P*	Primiparas (*n* = 58)			Multiparas (*n* = 46)		
					Exclusive breastfeeding at 3 months postpartum (*n* = 26)	Non-exclusive breastfeeding at 3 months postpartum (*n* = 32)	*p*	Exclusive breastfeeding at 3 months postpartum (*n* = 35)	Non-exclusive breastfeeding at 3 months postpartum (*n* = 11)	*p*
	Mean ± SD or n (%)	Mean ± SD or n (%)	Mean ± SD or n (%)		Mean ± SD or n (%)	Mean ± SD or n (%)		Mean ± SD or n (%)	Mean ± SD or n (%)	
Exclusive breastfeeding [n (%)]	52 (50.0)	47 (77.0)	5 (11.6)	< 0.001^a^	18 (69.2)	5 (15.6)	< 0.001^a^	29 (82.9)	0.(0.0)	< 0.001^b^
Presence of breast complications [n (%)]	45 (43.3)	19 (31.1)	26 (60.5)	0.003^a^	12 (46.2)	19 (59.4)	0.315^a^	7 (20.0)	7 (63.6)	0.010^b^
Breastfeeding self-efficacy scale^c^ [score]	45.8 ± 13.0	51.2 ± 11.6	38.1 ± 10.8	< 0.001	49.2 ± 11.0	39.1 ± 11.0	0.001	52.6 ± 11.5	35.3 ± 10.2	< 0.001
Perceived Stress Scale^d^ [score]	17.5 ± 6.5	16.6 ± 6.9	18.7 ± 5.8	0.173	16.4 ± 7.4	19.0 ± 5.5	0.129	16.7 ± 6.7	17.8 ± 6.6	0.626
Edinburgh Postnatal Depression Scale^e^ [score]	4.0 ± 3.7	3.4 ± 3.6	4.9 ± 3.6	0.006	4.2 ± 4.8	4.9 ± 3.6	0.516	2.8 ± 2.3	5.1 ± 3.9	0.019
Edinburgh Postnatal Depression Scale > 8 scores [n (%)]	11 (10.6)	6 (9.8)	5 (11.6)	0.759^b^	5 (19.2)	4 (12.5)	0.717^b^	1 (2.9)	1 (9.1)	0.425^b^
Postpartum Bonding Questionnaire^f^ [score]	9.4 ± 6.4	8.5 ± 6.9	10.6 ± 5.4	0.014	9.1 ± 8.3	9.9 ± 4.5	0.641	8.1 ± 5.8	12.7 ± 7.4	0.036
Support from husbands [n (%)]	63 (61.8)	38 (64.4)	25 (58.1)	0.520^a^	11 (44.0)	18 (56.3)	0.359^a^	27 (79.4)	7 (63.6)	0.421^b^
Support from parents [n (%)]	87 (83.7)	52 (88.1)	35 (81.4)	0.343^a^	21 (84.0)	29 (90.6)	0.687^b^	31 (91.2)	6 (54.5)	0.014^b^
Salivary cortisol levels before breastfeeding [μg/dL]	0.15 ± 0.09	0.13 ± 0.08	0.18 ± 0.10	< 0.001	0.13 ± 0.08	0.18 ± 0.12	0.037	0.13 ± 0.08	0.18 ± 0.06	0.045
Salivary cortisol levels after breastfeeding [μg/dL]	0.14 ± 0.08	0.12 ± 0.07	0.16 ± 0.09	0.013	0.12 ± 0.08	0.16 ± 0.09	0.098	0.12 ± 0.07	0.17 ± 0.09	0.062
Change in salivary cortisol levels between before and after breastfeeding [μg/dL]	−0.01 ± 0.07	−0.01 ± 0.04	−0.02 ± 0.09	0.126	−0.01 ± 0.05	−0.03 ± 0.10	0.376	0.00 ± 0.03	−0.01 ± 0.07	0.843
Percent change in salivary cortisol levels between before and after breastfeeding [%]	−0.03 ± 0.37	−0.001 ± 0.34	−0.01 ± 0.41	0.167	−0.040 ± 0.37	−0.07 ± 0.42	0.849	0.03 ± 0.32	−0.05 ± 0.36	0.468

*SD* Standard deviation

Mann-Whitney *U* test. ^a^Chi-square test. ^b^Fisher’s exact test

^c^The Japanese version of the Breastfeeding Self-Efficacy Scale Short Form, ^d^The Japanese version of the Perceived Stress Scale-10

^e^The Japanese version of Edinburgh Postnatal Depression Scale, ^f^The Japanese version of Postpartum Bonding Questionnaire

Multiple logistic regression analysis revealed the following factors as having significantly affected exclusive breastfeeding at 3 months postpartum: multiparity, having a university degree, no plan to return to work by 6 months postpartum, exclusive breastfeeding at 1 month postpartum, higher BSES-SF score at 1 month postpartum, and lower cortisol level after breastfeeding at 1 month postpartum in all the participants; exclusive breastfeeding and lower cortisol level after breastfeeding at 1 month postpartum in the primiparas; and lower cortisol level after breastfeeding, higher BSES-SF score, and absence of breast complications at 1 month postpartum in the multiparas (Table 3).

**Table 3:** Factors related to exclusive breastfeeding at 3 months postpartum

	All participants (*n* = 104)			Primiparas (*n* = 58)			Multiparas (*n* = 46)		
	AOR	(95% CI)	*p*	AOR	(95% CI)	*p*	AOR	(95% CI)	*p*
Parity (1: multipara, 0: primipara)	11.13	(2.08–59.59)	0.005	–			–		
Education levels (1: university or above, 0: others)	5.25	(1.04–26.53)	0.045	–			–		
Planning to return to work by 6 months postpartum (1: yes, 0: no)	0.02	(0.00–0.46)	0.014	–			–		
Exclusive breastfeeding at 1 month postpartum (1: yes, 0: no)	42.84	(6.05–303.52)	< 0.001	25.33	(4.75–134.98)	< 0.001	–		
Cortisol level after breastfeeding at 1 month postpartum (μg/dL)	0.00	(0.00–0.02)	0.007	0.00	(0.00–0.21)	0.019	0.00	(0.00–0.94)	0.047
Breastfeeding self-efficacy^a^ at 1 month postpartum (score)	1.07	(1.00–1.14)	0.036	–			1.18	(1.05–1.32)	0.007
Breast complications (1: presence, 0: absence)	–			–			0.09	(0.01–0.82)	0.033

Multiple logistic regression analysis, Backward Elimination (Wald)

Hosmer&Lemeshow: 0.459 (All participants), 0.742 (Primiparas), and 0.828 (Multiparas)

Of 104 women, 61 reported exclusive breastfeeding at 3 months postpartum

*AOR* Adjusted Odds Ratio, *CI* Confidence Interval

^a^The Japanese version of the Breastfeeding Self-Efficacy Scale Short Form

Factors associated with exclusive breastfeeding at 1 month postpartum were multiparity (*p* = 0.018), higher rates of natural childbirth (*p* = 0.001), lower rates of breast complications (*p* = 0.001), higher BSES-SF scores (*p* < 0.001), lower EPDS scores (*p* = 0.006), lower J-PBQ scores (*p* = 0.039), and support from husbands (*p* = 0.037) (data not shown). Salivary cortisol levels both before and after breastfeeding were not cross-sectionally associated with exclusive breastfeeding at 1 month postpartum.

## Discussion

The primary findings of our study were that lower salivary cortisol levels after breastfeeding, higher breastfeeding self-efficacy, and absence of breast complications at 1 month postpartum were possible modifiable predictors of exclusive breastfeeding at 3 months postpartum.

Lower cortisol levels after breastfeeding were associated with subsequent exclusive breastfeeding practices. Notably, the relationship was observed despite no cross-sectional relationship observed between cortisol levels and exclusive breastfeeding at 1 month postpartum. Cortisol levels usually decrease after breastfeeding as oxytocin inhibits cortisol secretion in women without mood distress [25]. Nevertheless, more than 40% of our participants had increased cortisol levels after breastfeeding than before. High cortisol levels after breastfeeding despite oxytocin action seem to reflect physiological and psychological stress related to breastfeeding. Previous studies reported no relationship between perceived stress and milk volume, although oxytocin levels were found to be decreased in women with psychological stress compared to the levels in those without psychological stress [12, 26]. Thus, we hypothesize that breastfeeding-related stress affects subsequent exclusive breastfeeding practice through psychological burden and associated behavioral changes, but not through fundamental physiological changes in lactation such as milk volume reduction. A stress response after breastfeeding is a modifiable factor. Approaches to stress reduction including prevention of breast complications, anxiety relief by providing advice about breastfeeding concerns, and relaxation therapy during breastfeeding could be effective [27]. In addition, maternal cortisol responses to breastfeeding vary according to the function of the CD38 rs3796863 [28], an ectoenzyme that mediates the release of oxytocin. A recent study has indicated that the CD38 rs3796863 CC genotype is associated with reduced oxytocin release during breastfeeding and, accordingly, fewer cortisol-reducing responses to breastfeeding [29]. The specific gene influences the association between breastfeeding and its cortisol responses through reduced oxytocin secretion, not through breastfeeding-related psychological stress. Thus, an association between post-breastfeeding cortisol levels and subsequent breastfeeding might be attenuated by not considering the effects of the gene. Further studies are needed to clarify the associations.

A higher BSES-SF score was associated with subsequent exclusive breastfeeding in the present study, as reported by a previous study of another population [30]. Breastfeeding self-efficacy reflects a mother’s confidence in her ability to breastfeed her infant. The BSES-SF contains the following items “I can always keep wanting to breastfeed” and “I can always be satisfied with my breastfeeding experience” [10, 19]. Women with positive answers to such items seemed less stressed about breastfeeding. Women with lower breastfeeding self-efficacy are more likely to perceive milk insufficiency [10, 31]. Such a perception has been described as a factor related to cessation of exclusive breastfeeding [32]. The perception of milk insufficiency itself was not assessed in our study because this concept was supposed to be contained in questions of the BSES-SF. A further detailed study regarding the relationship between breastfeeding self-efficacy, perception of milk insufficiency, and subsequent exclusive breastfeeding might contribute to the suggestion of concrete intervention methods for increasing the rate of exclusive breastfeeding. In the analyses according to parity, the association between breastfeeding self-efficacy and exclusive breastfeeding was observed only in multiparas. A previous study showed that the effect of self-efficacy on breastfeeding practice was much stronger in multiparas than primiparas [33]; this was because breastfeeding experiences in primiparas were affected more by subjective norms and social environment than by breastfeeding self-efficacy [33]. Therefore, the effectiveness of improving breastfeeding self-efficacy on breastfeeding practices might differ according to parity. A systematic review showed that prenatal and postpartum interventions focusing on improving breastfeeding self-efficacy lead to exclusive breastfeeding [34]. However, in Japan, the effects of prenatal intervention using a breastfeeding self-efficacy workbook are limited and effective only in baby-friendly hospitals and during the early postpartum period [35]. The development of more effective approaches taking parity into consideration are needed for postpartum Japanese women, based on the breastfeeding self-efficacy theoretical framework by Dennis [20]; for instance, the elements such as performance accomplishments, vicarious experience, verbal persuasion, and physiological and emotional states in the theoretical framework would help in the development of self-efficacy expectations [20].

In multiparas, absence of breast complications at 1 month postpartum was associated with subsequent exclusive breastfeeding. Although this association supported the findings of previous studies [32, 36], we did not ascertain the reason why it was observed only in multiparas. In general, primiparas experience more breast complications than multiparas [37], as was also observed in the present study. Furthermore, breast complications strongly relate to psychological breastfeeding-related stress [8]. The strength of the association between the presence of breast complications, breastfeeding-related stress, and exclusive breastfeeding practice may depend on participant characteristics and individual stress responses associated with breast complications. Some of the recommended management strategies for women who consider breastfeeding cessation because of breast complications, based on the breastfeeding pain reasoning model, include improving healing, improving the attachment of infant to the breast, maximizing comfortable positions for feeding, and psychological support [38]. Self-management intervention for these strategies would be essential to improve breast complications and relieve breastfeeding-related stress [39].

As with previous studies, parity and education level were associated with exclusive breastfeeding in our study [4, 13]. However, the effect of parity on exclusive breastfeeding practice is not a simple correlation; rather, it often varies by study populations and previous breastfeeding experiences in multiparous women [5, 40, 41]. Women with higher education levels easily access health-related information and have more favorable attitudes toward breastfeeding [42, 43]. Such behavioral characteristics in women with higher education levels might help them achieve exclusive breastfeeding.

Planning to return to work by 6 months postpartum was associated with less exclusive breastfeeding at 3 months postpartum in our study. Postpartum women who planned to return to work in the earlier postpartum period tended to select partial breastfeeding or formula feeding [31, 44]. In Japan, continuing exclusive breastfeeding is difficult for some working women because of the work environment and nursery policies. A private space and consideration for expressing breast milk during working hours are often lacking, although many companies have been attempting to provide such environments. Nursery policies often refuse breast milk storage due to hygienic reasons. Such situations sometimes make women discontinue exclusive breastfeeding [45]. In addition, partners’ attitudes against breastfeeding and childcare are possible important elements that prevent women from discontinuing breastfeeding after returning to work [46]. Environmental and emotional support from family members, employers, and nursery staff members is essential for working women to continue exclusive breastfeeding.

Exclusive breastfeeding at 1 month postpartum is a strong predictor of the same at 3 months postpartum. However, even if women are not exclusively breastfeeding at 1 month postpartum, they may change to exclusive breastfeeding later on, as observed in the present study. The first 3 months after childbirth is a critical period for the establishment of exclusive breastfeeding [47]. Thus, providing medical and psychological support is crucial during this period. Stress levels after breastfeeding, breastfeeding self-efficacy, and breast complications are possible key modifiable predictors. In Japan, women have state-funded medical checkups at 1 month postpartum. Thereafter, if they desire medical care regarding breastfeeding, they have to access it by themselves. By 1 month postpartum, healthcare providers’ advice and intervention for reducing breastfeeding-related stress responses, improving breastfeeding self-efficacy, and preventing breast complications may be effective in establishing and continuing exclusive breastfeeding. Further intervention studies are required to confirm the effectiveness.

The present study had two limitations. First, the dropout rate was higher than expected. Although no differences in infant feeding modality and salivary cortisol levels between dropouts and participants were found, the high dropout rate may have affected the relationship between cortisol levels and breastfeeding practice observed in the study. Second, we could not follow-up the participants for a full 6 months postpartum, although exclusive breastfeeding for 6 months is recommended. However, the key strength of this study is that we demonstrated the relationship between stress responses associated with breastfeeding and subsequent exclusive breastfeeding, using objective measures of stress.

## Conclusions

Breastfeeding-related stress levels at 1 month postpartum were identified as a possible modifiable predictor of exclusive breastfeeding at 3 months postpartum, regardless of parity. In multiparas, breastfeeding self-efficacy and presence of breast complications at 1 month postpartum could be possible modifiable factors. Further research is needed to determine whether approaches to reducing breastfeeding-related stress, improving breastfeeding self-efficacy, and preventing breast complications might be effective in increasing exclusive breastfeeding practices.

## Data Availability

The datasets used and analyzed during the current study are available from the corresponding author on reasonable request.

## Abbreviations

BSES-SF: Breastfeeding Self-Efficacy Scale Short Form
PSS-10: Perceived Stress Scale-10
EPDS: Edinburgh Postnatal Depression Scale
J-PBQ: The Japanese version of Postpartum Bonding Questionnaire

## References

[1] World Health Organization (2011). Exclusive breastfeeding for six months best for babies everywhere.

[2] Mothers’ and Children’s Health Organization Co., Ltd (2017). Nutritional status by infant age. Maternal and child health statistics of Japan.

[3] Ministry of Health, Labour and Welfare (2016). National nutrition survey on preschool children, 2015.

[4] Cohen SS, Alexander DD, Krebs NF, Young BE, Cabana MD, Erdmann P (2018). Factors associated with breastfeeding initiation and continuation: a meta-analysis. J Pediatr.

[5] Whipps MD (2017). Education attainment and parity explain the relationship between maternal age and breastfeeding duration in U.S. mothers. J Hum Lact.

[6] Holowko N, Jones M, Koupil I, Tooth L, Mishra G (2016). High education and increased parity are associated with breast-feeding initiation and duration among Australian women. Public Health Nutr.

[7] Adugna B, Tadele H, Reta F, Berhan Y (2017). Determinants of exclusive breastfeeding in infants less than six months of age in Hawassa, an urban setting, Ethiopia. Int Breastfeed J.

[8] Abou-Dakn M, Schäfer-Graf U, Wöckel A (2009). Psychological stress and breast diseases during lactation. Breastfeed Rev.

[9] Odom EC, Li R, Scanlon KS, Perrine CG, Grummer-Strawn L (2013). Reasons for earlier than desired cessation of breastfeeding. Pediatrics.

[10] Otsuka K, Dennis CL, Tatsuoka H, Jimba M (2008). The relationship between breastfeeding self-efficacy and perceived insufficient milk among Japanese mothers. J Obstet Gynecol Neonatal Nurs.

[11] Maehara K, Mori E, Iwata H, Sakajo A, Aoki K, Morita A (2017). Postpartum maternal function and parenting stress: comparison by feeding methods. Int J Nurs Pract.

[12] Ueda T, Yokoyama Y, Irahara M, Aono T (1994). Influence of psychological stress on suckling-induced pulsatile oxytocin release. Obstet Gynecol.

[13] Kaneko A, Kaneita Y, Yokoyama E, Miyake T, Harano S, Suzuki K (2006). Factors associated with exclusive breastfeeding in Japan: for activities to support child-rearing with breastfeeding. J Epidemiol.

[14] Al-Safi ZA, Polotsky A, Chosich J, Roth L, Allshouse AA, Bradford AP (2018). Evidence for disruption of normal circadian cortisol rhythm in women with obesity. Gynecol Endocrinol.

[15] Kusaka M, Matsuzaki M, Shiraishi M, Haruna M (2016). Immediate stress reduction effects of yoga during pregnancy: one group pre–post test. Women Birth.

[16] Mimura C, Griffiths PA (2008). Japanese version of the perceived stress scale: cross-cultural translation and equivalence assessment. BMC Psychiatry.

[17] Okano T, Murata M, Masuji F, Tamaki R, Nomura J, Miyaoka H (1996). Validation and reliability of Japanese version of EPDS (Edinburgh Postnatal Depression Scale). Arch Psychiatr Diagn Clin Eval.

[18] Suetsugu Y, Honjo S, Ikeda M, Kamibeppu K (2015). The Japanese version of the postpartum bonding questionnaire: examination of the reliability, validity, and scale structure. J Psychosom Res.

[19] Dennis CL (2003). The breastfeeding self-efficacy scale: psychometric assessment of the short form. J Obstet Gynecol Neonatal Nurs.

[20] Dennis CL (1999). Theoretical underpinnings of breastfeeding confidence: a self-efficacy framework. J Hum Lact.

[21] Bandura A (1977). Self-efficacy: toward a unifying theory of behavioral change. Psychol Rev.

[22] Cohen S, Kamarck T, Mermelstein R (1983). A global measure of perceived stress. J Health Soc Behav.

[23] Cox JL, Holden JM, Sagovsky R (1987). Detection of postnatal depression. Development of the 10-item Edinburgh postnatal depression scale. Br J Psychiatry.

[24] Brockington IF, Fraser C, Wilson D (2006). The postpartum bonding questionnaire: a validation. Arch Womens Ment Health.

[25] Cox EQ, Stuebe A, Pearson B, Grewen K, Rubinow D, Meltzer-Brody S (2015). Oxytocin and HPA stress axis reactivity in postpartum women. Psychoneuroendocrinology.

[26] Hill PD, Aldag JC, Chatterton R, Zinaman M (2005). Psychological distress and milk volume in lactating mothers. West J Nurs Res.

[27] Shukri NHM, Wells J, Eaton S, Mukhtar F, Petelin A, Jenko-Pražnikar Z (2019). Randomized controlled trial investigating the effects of a breastfeeding relaxation intervention on maternal psychological state, breast milk outcomes, and infant behavior and growth. Am J Clin Nutr.

[28] Krol KM, Monakhov M, Lai PS, Ebstein RP, Heinrichs M, Grossmann T (2018). Genetic variation in the maternal oxytocin system affects cortisol responsiveness to breastfeeding in infants and mothers. Adapt Hum Behav Physiol.

[29] Jin D, Liu HX, Hirai H, Torashima T, Nagai T, Lopatina O (2007). CD38 is critical for social behaviour by regulating oxytocin secretion. Nature.

[30] Linares AM, Rayens MK, Dozier A, Wiggins A, Dignan MB (2015). Factors influencing exclusive breastfeeding at 4 months postpartum in a sample of urban Hispanic mothers in Kentucky. J Hum Lact.

[31] Gökçeoğlu E, Küçükoğlu S (2017). The relationship between insufficient milk perception and breastfeeding self-efficacy among Turkish mothers. Glob Health Promot.

[32] Gianni ML, Bettinelli ME, Manfra P, Sorrentino G, Bezze E, Plevani L (2019). Breastfeeding difficulties and risk for early breastfeeding cessation. Nutrients.

[33] Bartle NC, Harvey K (2017). Explaining infant feeding: the role of previous personal and vicarious experience on attitudes, subjective norms, self-efficacy, and breastfeeding outcomes. Br J Health Psychol.

[34] Brockway M, Benzies K, Hayden KA (2017). Interventions to improve breastfeeding self-efficacy and resultant breastfeeding rates: a systematic review and meta-analysis. J Hum Lact.

[35] Otsuka K, Taguri M, Deniss CL, Wakutani K, Awano M, Yamaguchi T (2014). Effectiveness of a breastfeeding self-efficacy intervention: do hospital practices make a difference?. Matern Child Health J.

[36] Wagner EA, Chantry CJ, Dewey KG, Nommsen-Rivers LA (2013). Breastfeeding concerns at 3 and 7 days postpartum and feeding status at 2 months. Pediatrics.

[37] Feenstra MM, Kirkeby MJ, Thygesen M, Danbjørg DB, Kronborg H (2018). Early breastfeeding problems: a mixed method study of mothers’ experiences. Sex Reprod Health.

[38] Amir LH, Jones LE, Buck ML (2015). Nipple pain associated with breastfeeding: incorporating current neurophysiology into clinical reasoning. Aust Fam Physician.

[39] Lucas R, Zhang Y, Walsh SJ, Evans H, Young E, Starkweather A (2019). Efficacy of a breastfeeding pain self-management intervention: a pilot randomized controlled trial. Nurs Res.

[40] Wu X, Gao X, Sha T, Zeng G, Liu S, Li L (2019). Modifiable individual factors associated with breastfeeding: a cohort study in China. Int J Environ Res Public Health.

[41] Huang Y, Ouyang YQ, Redding SR (2019). Previous breastfeeding experience and its influence on breastfeeding outcomes in subsequent births: a systematic review. Women Birth.

[42] Ueno M, Ohara S, Inoue M, Tsugane S, Kawaguchi Y (2012). Association between education level and dentition status in Japanese adults: Japan public health center-based oral health study. Community Dent Oral Epidemiol.

[43] Sittlington J, Stewart-Knox B, Wright M, Bradbury I, Scott JA (2007). Infant-feeding attitudes of expectant mothers in Northern Ireland. Health Educ Res.

[44] Ohta Y, Sugawara S (2006). Factors of decision-making to discontinue breast-feeding. Med J Sendai R C H.

[45] Uehara K, Kawasaki K, Usui A (2009). The breast-feeding environment for working mothers in O city. J Jpn Soc Breastfeed Res.

[46] Tsai SY (2014). Influence of partner support on an employed mother's intention to breastfeed after returning to work. Breastfeed Med.

[47] Hashizume Y, Horigome K, Nameda T (2018). Breastfeeding concerns among first-time mothers―based on the results of interviews with mothers who experienced breastfeeding difficulties and concerns in 4 months after hospital discharge―. J Jpn Acad Midwif.

